# Hierarchical Beamforming Optimization for ISAC-Enabled RSU Systems in Complex Urban Environments

**DOI:** 10.3390/s25216803

**Published:** 2025-11-06

**Authors:** Zhiyuan You, Na Lv, Guimei Zheng, Xiang Wang

**Affiliations:** 1 The Graduate School, Air Force Engineering University, Xi’an 710038, China; yzy343625419@163.com; 2 College of Information and Navigation, Air Force Engineering University, Xi’an 710038, China; 3 Air Defense and Antimissile School, Air Force Engineering University, Xi’an 710038, China

**Keywords:** integrated sensing and communications, beamforming, hierarchical optimization, multi-user interference, semidefinite program

## Abstract

Integrated Sensing and Communication (ISAC)-enabled Roadside Units (RSUs) encounter significant performance trade-offs between target sensing and multi-user communication in complex urban environments, where conventional optimization methods are prone to converging to local optima and joint optimization methods often yield sub-optimal results due to conflicting objectives. To address the challenge of trade-off between sensing and communication performance, this paper proposes a hierarchical beamforming optimization solution designed to tackle joint sensing–communication problems in such scenarios. The overall optimization problem is decomposed into a two-level “leader-follower” structure. In the leader layer, we introduce a max–min strategy based on the bisection method to transform the non-convex Signal-to-Interference-plus-Noise Ratio (SINR) optimization problem into a second-order cone constraint problem and solve the communication beamforming vector. In the follower layer, the Signal-to-Clutter-plus-Noise Ratio (SCNR) maximization problem is converted into a Semi-Definite Programming (SDP) problem solved via the CVX toolbox. Additionally, we introduce a “spatiotemporal resource isolation” mechanism to project the sensing beam onto the null space of the communication channel. The hierarchical optimization solution jointly optimizes communication SINR and sensing SCNR, enabling an effective balance between sensing accuracy and communication reliability. Simulation results demonstrate the proposed method’s effectiveness in simultaneously improving sensing accuracy and communication reliability.

## 1. Introduction

The advancement of 6G technology has brought Integrated Sensing and Communication (ISAC) to the forefront of Vehicle-to-Everything (V2X) research [1,2,3]. ISAC-enabled V2X systems facilitate comprehensive data exchange between vehicles and their environment, including infrastructure, pedestrians, and networks [4,5,6]. To optimize urban road management efficiency, Simultaneous Localization and Mapping (SLAM) technology is increasingly being integrated with ISAC [7]. While traditional SLAM implementations primarily rely on vision and LiDAR sensors, recent breakthroughs have shown that wireless channel propagation characteristics can be used to construct highly accurate 2D environmental maps [8,9]. This synergy establishes a novel co-design paradigm for ISAC-SLAM integration in V2X. Recent research demonstrate that an ISAC base station (BS) can effectively function as a roadside unit (RSU) [2,10,11]. These advanced ISAC-RSUs enhance environmental sensing and establish real-time SLAM capabilities through low-latency V2X links, compensating for onboard sensor limitations. Smart city implementations have validated this approach, with [12] demonstrating that MIMO radar-equipped RSUs can utilize echo signals for channel estimation and adaptive beamforming. This enables ISAC-RSU as the core infrastructure for future 6G vehicular networks. In dense urban environments, buildings and other obstacles can significantly impede signal propagation. Relay communication effectively extends coverage and ensures continuous connectivity between vehicles and infrastructure by mitigating the impact of physical obstructions. In high-density traffic scenarios, relay nodes facilitate multi-path data transmission, enhancing communication reliability and stability through redundancy and improved link diversity [2]. By distributing the communication load across multiple nodes, relay systems increase overall network capacity, which is crucial for supporting the concurrent communication demands of numerous connected vehicles and devices [13]. Ref. [10] has shown that RSUs, as critical components of next-generation urban communication infrastructures, possess significant capabilities in directional relay-assisted transmission. Specifically, these ISAC-RSUs can simultaneously sense urban road conditions and function as relay base stations, transmitting directional communication signals to user equipment (UE) [14]. Furthermore, Ref. [15] introduced a hybrid reconfigurable intelligent surface-assisted downlink ISAC system that offers a new reference approach for integrating RSUs and reconfigurable intelligent surfaces to enhance UE communication rates. Refs. [16,17] propose deep learning (DL)-based predictive beamforming for ISAC in vehicular networks and demonstrate DL’s potential for next-generation vehicular ISAC systems.

However, the simultaneous delivery of high-quality communication and precise detection becomes particularly challenging in complex multi-user, multi-target scenarios. Balancing communication-centric and sensing-centric objectives remains a critical issue in ISAC systems due to competing demands for spectral resources, beamforming design, and signal processing strategies [18]. Furthermore, RSUs face substantial signal interference that adversely affects performance in both communication and sensing domain. In communication, downlink signals from adjacent RSUs generate multiple access interference (MAI) with UEs, while spatiotemporal resource reuse further aggravates multi-user interference (MUI) [19]. In sensing, the overlap of RSU echo signals in the time–frequency domain can degrade the signal-to-noise ratio (SNR). These dual-domain interference problems necessitate advanced beamforming solutions for ISAC-RSU systems. Additionally, urban multi-path effects and building occlusions constrain their detection capabilities, as sensing beams reflect off buildings, creating clutter that interferes with the ISAC-RSU receiver. These challenges require the development of enhanced beamforming designs for ISAC-RSU systems [20,21,22,23].

Contemporary studies demonstrate that beamforming design plays a crucial role in establishing the Pareto frontier, which quantifies the fundamental trade-off between sensing and communication performance in ISAC systems. ISAC beamforming aims to achieve optimal performance trade-offs while suppressing mutual interference between sensing and communication signals [24]. In the context of ISAC beamforming methods, Refs. [16,17] address dynamic channel variations caused by high mobility, leveraging neural networks to forecast beamforming vectors and optimize ISAC performance. However, the internal weights of its network are difficult to map to physical-layer parameters (such as beam pointing angle and null notch depth), limiting their effectiveness in fault diagnosis. In contrast, conventional methods offer closed-form solutions derived from analytical algorithms based on channel state information, thereby facilitating optimization and parameter adjustment. Ref. [25] investigated a weighted minimum mean square error (WMMSE) ISAC solution based on mutual information (MI) to transform a non-convex problem into a convex one, utilizing MI as a metric to assess both sensing and communication performance. Furthermore, cell-free ISAC MIMO systems represent a significant advancement in the simultaneous provision of communication services to users and the detection of targets [26,27,28,29]. Specifically, Ref. [26] proposed a joint beamforming design for cell-free ISAC MIMO systems, where distributed MIMO access points collaboratively facilitate communication for user equipment (UEs) while simultaneously sensing targets. This study employs a max–min fairness formulation and derives the optimal structure of beamforming vectors to suppress both MAI and MUI, thereby improving the communication SINR [27]. Additionally, in low-altitude urban environments, objects exhibiting a significant radar cross-section (RCS), including structures and metallic advertisements, can generate scattered clutter patches that interfere with the receiver’s performance. In order to address the issue of environmental clutter, Ref. [29] proposed a constant-modulus waveform design method for ISAC systems by incorporating cyclic optimization, Dinkelbach’s transform, and the ADMM algorithm. This method effectively addresses the non-convex optimization problem while optimizing both radar detection performance and communication service quality, but it suffers from high computational complexity due to excessive matrix operations and exhibits relatively slow convergence rates.

In response to these issues, we propose a dual-base-station ISAC system with a transmitting RSU and a receiving RSU, suitable for road surveillance and directional relay communication in urban areas. The BS transmits downlink signals to the ISAC-RSU, providing high-quality services. We propose a hierarchical optimization solution for ISAC beamforming, specifically designed for ISAC-RSU systems operating in complex environments. The main contributions of this paper are as follows:

We construct a hierarchical and collaborative ISAC beamforming optimization framework, and it has the potential to overcome the limitations imposed by the direct coupling of sensing and communication objectives in existing single-stage optimization approaches. The leader layer prioritizes ensuring communication performance for multiple UEs by adopting a max–min fairness strategy to optimize the SINR. The communication beamforming vector is derived using the bisection method in conjunction with second-order cone programming, thereby guaranteeing that the communication links satisfy both fairness and reliability requirements across UEs. Under the constraint of fixed communication beamforming parameters from the follower layer, the problem of maximizing the sensing SCNR is formulated as a semi-definite programming (SDP) problem, enabling collaborative design of the sensing beam through convex optimization techniques. Therefore, it achieves a coherent synergy by prioritizing communication performance to precisely enhance sensing performance, in contrast to the performance trade-offs inherent in traditional approaches. Finally, we utilize the sensing and communication (S&C) MI rate as a metric to evaluate whether the beamforming solution can effectively balance sensing and communication performance.

Notations: Bold letters denote vectors and matrices. Italicized letters denote variables. ${\left[\cdot \right]}^{T}$ and ${\left[\cdot \right]}^{H}$ denote the transpose operation and the Hermitian operation, respectively. ${\mathbb{C}}^{N\times N}$ denotes a N×N complex matrix. Re[*a*] denotes the real part of a scalar *a*. tr(A) denotes the trace of a matrix ***A***. E[⋅] denotes the expectation operation. $\left\|a\right\|$ and ${\left\|A\right\|}_{F}$ denote the *l*_2_-norm of ***a*** and the Frobenius norms of ***A***. $\mathrm{CN}(0,\;\sigma^{2})$. represents a circularly symmetric complex Gaussian random variable with zero mean and variance $\sigma^{2}$.

## 2. System Model

**Figure 1:**
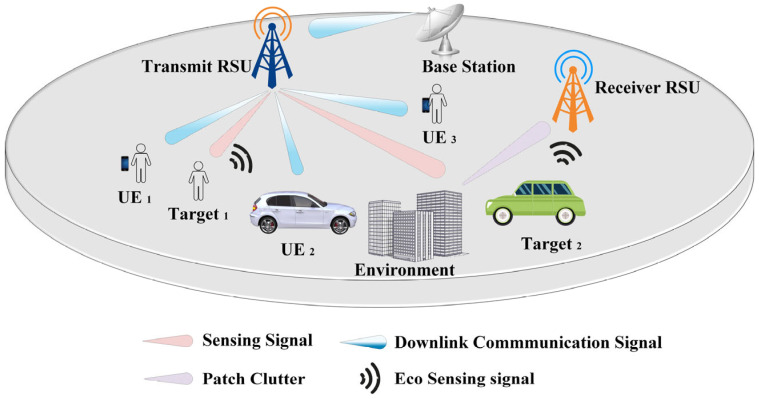
ISAC-RSU system model using beamforming.

In this study, we consider a bistatic ISAC-RSU system comprising a transmit RSU and a receive RSU. Each unit is equipped with a half-wavelength-spaced uniform linear array (ULA), consisting of $N_{t}$ transmit antennas and $N_{r}$ receive antennas, respectively. The system configuration is illustrated in Figure 1. The transmit RSU emits *S* sensing streams toward targets and *U* communication streams toward UEs in the downlink, enabling simultaneous data transmission and sensing. The targets reflect the transmitted sensing signals, generating echo returns that are captured by the receive RSU. Additionally, the BS transmits a downlink communication signal to the ISAC transmitter, carrying relayed information intended for the UEs. It is assumed that the receive RSU also receives sensing clutter originating from *C* propagation paths in the surrounding environment.

For the sake of simplicity, we consider that the transmit RSU is equipped with digital beamforming capabilities, which enable us to proceed with the establishment of the signal model.

### 2.1. Signal Model

This sub-section introduces the ISAC transmitted signal model, which is specifically designed to enable target sensing while simultaneously supporting relay communication with UEs. The ISAC transmitter is capable of transmitting *U* communication streams to cooperative users and *S* sensing streams to non-cooperative targets. $x_{u}[l]\in \mathbb{C}$ and $x_{s}[l]\in \mathbb{C}$ denote the transmitted communication signal and the transmitted sensing signal for the *l*-th symbol, respectively. The data symbols of these streams can be accommodated within a fast time snapshot of the ISAC transmitter. The transmitted signal is a linear combination of the communication and sensing signals. Therefore, we exploit the waveform diversity capability of the MIMO ISAC system to transmit distinct signals in specific directions. The transmitted signal $x[l]\in {\mathbb{C}}^{N_{t}}$ is expressed as follows:

(1) $$x[l]=\sum_{u=1}^{U}w_{u}x_{u}[l]+\sum_{s=1}^{S}w_{s}x_{s}[l]=\sum_{n=1}^{N}w_{n}x_{n}[l],$$

where $w_{u}$ and $w_{s}$ denote transmit beamforming vectors for the *u*-th user and for the *s*-th target, respectively, N=U+S denotes the aggregate number of the communication and sensing streams sent by the transmitter, and $w_{n}\in {\mathbb{C}}^{N_{t}}$ denotes the transmit beamforming vector included $w_{u}$ and $w_{s}$.

We assume that there are *L* symbols in the signal; then, Equation (1) can be expressed as

(2) $$X=\bar{W}\bar{X}\in {\mathbb{C}}^{N_{t}\times L},$$

(3) $$\bar{W}=[w_{1},w_{2},\ldots ,w_{N}]\in {\mathbb{C}}^{N_{t}\times N},$$

(4) $$\bar{X}={\left[x_{1},x_{2},\ldots ,x_{N}\right]}^{T}\in {\mathbb{C}}^{N\times L},$$

where $\bar{W}$ denotes the transmitted beamforming matrix, and $\bar{X}$ denotes the transmitted symbol sequence matrix; the transmit sequence vector of *L* symbols for each stream is denoted as

(5) $$x_{n}={\left[x_{n}[1],x_{n}[2],\ldots ,x_{n}[L]\right]}^{T}.$$

In this analysis, we assume that the messages carried by the sensing and communication signals are statistically independent and that the sensing signals can be generated using pseudo-random coding. Furthermore, we assume that the symbols have unit average energy, $E[{\left|x_{n}[l]\right|}^{2}]=1$.

### 2.2. Communication Model

We represent $h_{u}\in {\mathbb{C}}^{1\times N_{t}}$ as the communication stationary channel from the transmitter to the *u*-th user, excluding non-line-of-sight (N-LOS) channels from consideration. The communication channel matrix $H_{c}$ can be expressed as

(6) $$H_{c}={\left[h_{1},h_{2},\ldots ,h_{U}\right]}^{T}\in {\mathbb{C}}^{U\times N_{t}}.$$

We assume that the communication channel is a steering matrix from the transmitter to the users, i.e., $H_{c}=A_{u}^{T}$. The steering matrix is given by

(7) $$A_{u}=[a(\theta_{1}),a(\theta_{2}),\ldots ,a(\theta_{U})],$$

where $a(\theta_{u})=[1,e^{j\pi \sin\theta_{u}},\ldots ,e^{j\pi (N_{t}-1)\sin\theta_{u}}]$ denotes the steering vector, and $\theta_{u}$ represents the azimuth angle from the transmitter to the *u*-th user. Thus, the communication channel matrix $H_{c}={\left[a(\theta_{1}),a(\theta_{2}),\ldots ,a(\theta_{U})\right]}^{T}$.

For the communication signals received by users, it is assumed that the channel remains constant over the transmission of *L* consecutive symbols. The signal received by each user consists of five components: the desired communication signal, MUI, sensing interference, BS communication interference, and noise. The received signal at the *u*-th user can be expressed as

(8) $$\begin{array}{lll} y_{u}[l] & = & h_{u}x[l]+h_{u}w_{g}x_{g}[l]+n_{u}[l]\\ & = & h_{u}w_{u}x_{u}[l]+\sum_{u\prime \in U/\{u\}}h_{u}w_{u\prime }x_{u\prime }[l]+\sum_{s=1}^{S}h_{u}w_{s}x_{s}[l]\\ & & +h_{u}w_{g}x_{g}[l]+n_{u}[l], \end{array}$$

where ={1,2,…,U}, $w_{g}$ denotes transmit precoder vector of the BS to the transmit RSU, $x_{g}[l]$ denotes transmit symbol signal of the BS to the transmit RSU, and $n_{u}[l]\sim (0,\sigma_{u}^{2})$ denotes the receiver noise of *u*-th user. Notably, since $h_{u}w_{g}x_{g}[l]$ represents the signal transmitted from the BS to the RSU, it acts as an interference signal for the UE.

Due to the BS’s supplementary role in relaying the communication stream to the transmit RSU, it is equipped with a ULA of $N_{t}$ transmit antennas spaced at half-wavelength intervals, dedicated to communication. When the BS transmits only the communication stream to the transmit RSU, the beamforming vector $w_{g}$ can be articulated as follows:

(9) $$w_{g}=\sqrt{p_{g}}a_{g},$$

where $p_{g}$ denotes the beamforming power, $a_{g}=[1,e^{j\pi \sin\theta_{g}},\ldots ,e^{j\pi (N_{t}-1)\sin\theta_{g}}]\in {\mathbb{C}}^{1\times N_{t}}$ denotes communication channel from BS to transmit RSU, and $\theta_{g}$ represents the azimuth angle from the BS to the transmit RSU.

Upon referring to Equation (8), it can be deduced that the desired communication signal received by the *u*-th user is denoted as $h_{u}w_{u}x_{u}[l]$, while the remaining signals are classified as interferences and noise. Consequently, the SINR of the received signal is used as a metric for evaluating communication performance. The SINR for the receiver associated with the *u*-th user can be expressed as

(10) $$\begin{array}{ll} {\mathrm{SINR}}_{u} & =\frac{E[{\left|h_{u}w_{u}x_{u}[l]\right|}^{2}]}{E[{\left|\sum_{u\prime \in U/\{u\}}h_{u}w_{u\prime }x_{u\prime }[l]\right|}^{2}]+E[{\left|\sum_{s=1}^{S}h_{u}w_{s}x_{s}[l]\right|}^{2}]+E[{\left|h_{u}w_{g}x_{g}[l]\right|}^{2}]+E[{\left|n_{u}[l]\right|}^{2}]}\\ & =\frac{{\left|h_{u}w_{u}\right|}^{2}}{{\left|\sum_{u\prime \in U/\{u\}}h_{u}w_{u\prime }\right|}^{2}+{\left|\sum_{s=1}^{S}h_{u}w_{s}\right|}^{2}+{\left|h_{u}w_{g}\right|}^{2}+\sigma_{u}^{2}}. \end{array}$$

### 2.3. Sensing Model

For the sensing model, we assume the existence of *C* clutter patches within the environment and take into account the availability of two sensing channels: $G_{s}$ (transmit RSU–targets–receive RSU) and $G_{c}$ (transmit RSU–clutter patch–receive RSU), given by

(11) $$G_{s}=\alpha_{s}a(\phi_{s})a^{H}(\varphi_{s}),$$

(12) $$G_{c}=\alpha_{c}a(\phi_{c})a^{H}(\varphi_{c}),$$

where $G_{s}\in {\mathbb{C}}^{N_{r}\times N_{t}}$ and $G_{c}\in {\mathbb{C}}^{N_{r}\times N_{t}}$ denote the beam-space sensing channel matrices corresponding to the target and clutter, respectively, $\alpha_{s}\sim \mathrm{CN}(0,\zeta_{s}^{2})$, denotes the sensing target channel gain, and $\alpha_{c}\sim \mathrm{CN}(0,\zeta_{c}^{2})$ denotes the sensing clutter channel gain. $\phi_{s}$ and $\phi_{c}$ denote the direction of arrival (DOA) of the targets and clutter patches, respectively, as observed at the receive RSU. $\varphi_{s}$ and $\varphi_{c}$ denote the direction of departure (DOD) from the transmit RSU toward the targets and clutter patches, where S={1,2,…,S}.

It is worth noting that we employ the Swerling-I model for the beam-space sensing channel, which assumes slow fluctuations in the RCS. Consequently, the sensing channel remains stable during the transmission of *L* sensing and communication symbols. The signal received at the receive RSU at time instant *l* is given by

(13) $$\begin{array}{ll} y[l] & =\sum_{s=1}^{S}G_{s}x[l]+\sum_{c=1}^{C}G_{c}x[l]+n_{s}[l]\\ & =\sum_{s=1}^{S}G_{s}(\sum_{u=1}^{U}w_{u}x_{u}[l]+\sum_{s=1}^{S}w_{s}x_{s}[l])+\sum_{c=1}^{C}G_{c}(\sum_{n=1}^{N}w_{n}x_{n}[l])+n_{s}[l]\\ & =\sum_{s=1}^{S}G_{s}w_{s}x_{s}[l]+\sum_{s=1}^{S}G_{s}(\sum_{u=1}^{U}w_{u}x_{u}[l]+\sum_{s\prime \in S//\{s\}}w_{s\prime }x_{s\prime }[l])+\sum_{c=1}^{C}G_{c}(\sum_{n=1}^{N}w_{n}x_{n}[l])+n_{s}[l]. \end{array}$$

Assuming the presence of *L* symbols within the signal, Equation (13) can be reformulated as follows:

(14) $$\begin{array}{ll} Y= & \sum_{s=1}^{S}\alpha_{s}a(\phi_{s})a^{H}(\varphi_{s})W_{s}X_{s}+\sum_{s=1}^{S}\alpha_{s}a(\phi_{s})a^{H}(\varphi_{s})(\sum_{u=1}^{U}W_{u}X_{u}+\sum_{s\prime \in S/\{s\}}W_{s\prime }X_{s\prime })\\ & \;+\sum_{c=1}^{C}\alpha_{c}a(\phi_{c})a^{H}(\varphi_{c})(\sum_{n=1}^{N}W_{n}X_{n})+N_{s}, \end{array}$$

where the desired sensing signals received by the receive RSU are $\sum_{s=1}^{S}\alpha_{s}a(\phi_{s})a^{H}(\varphi_{s})W_{s}X_{s}$, while the other signals are classified as clutter, MUI, and noise. Subsequently, the signal-to-clutter-plus-noise ratio (SCNR) of the received signal is adopted as a metric for performance evaluation. The SCNR of the receiver can be obtained as

(15) $$\mathrm{SCNR}=\frac{E[{\left\|\sum_{s=1}^{S}\alpha_{s}a(\phi_{s})a^{H}(\varphi_{s})W_{s}X_{s}\right\|}_{F}^{2}]}{E[{\left\|\sum_{s=1}^{S}\alpha_{s}a(\phi_{s})a^{H}(\varphi_{s})(\sum_{u=1}^{U}W_{u}X_{u}+\sum_{s\prime \in S/\{s\}}W_{s\prime }X_{s\prime })+\right\|}_{F}^{2}]+E[{\left\|\sum_{c=1}^{C}\alpha_{c}a(\phi_{c})a^{H}(\varphi_{c})(\sum_{n=1}^{N}W_{n}X_{n})\right\|}_{F}^{2}]+E[{\left\|N_{s}\right\|}_{F}^{2}]}.$$

The numerator of the SCNR represents the mathematical expectation of the desired sensing signal, while the denominator includes the sum of the mathematical expectations of multi-user interference, clutter from patches, and noise. Notably, the incorporated clutter patch enhances the SCNR closed-form expression, allowing for a better representation of complex urban environments. This study focuses exclusively on optimizing ISAC transmission beamforming and does not address clutter mitigation techniques at the receive RSU.

## 3. A Hierarchical Optimization Solution for ISAC Beamforming

In this section, our objective is to obtain optimal communication beamforming vectors ${\left\{w_{u}\right\}}_{u\in U}$ and sensing beamforming vectors ${\left\{w_{s}\right\}}_{s\in S}$ by jointly optimizing communication SINR and sensing SCNR. We propose a hierarchical optimization solution for ISAC beamforming that enables a seamless transition from communication-centric to sensing-centric design, thereby facilitating progressive convergence toward a globally optimal solution. The complex joint optimization problem is decomposed into multiple interrelated sub-problems. Specifically, communication beamforming is formulated as leader-layer optimization, while sensing beamforming is addressed in follower-layer optimization, allowing for coordinated optimization toward the overall objective. Notably, this hierarchical decomposition optimization is conceptually analogous to bi-level formulations, with its primary innovation lying in structural organization rather than the introduction of a novel optimization principle.

### 3.1. A Maximize–Minimize Optimization Solution Based on the Bisection Method

For leader-layer optimization, our objective is to maximize the SINR, as higher SINR directly improves communication beamforming performance. Consequently, we adopt a max–min optimal strategy to achieve robust SINR enhancement and determine the associated beamforming variables ${\left\{w_{u}\right\}}_{u\in U}$. The problem can be formulated as

(16) $$\underset{\left\{w_{u}\right\}}{\max}\;\underset{\left\{u\right\}}{\min}\frac{{\left|h_{u}w_{u}\right|}^{2}}{{\left|\sum_{u\prime \in U/\{u\}}h_{u}w_{u\prime }\right|}^{2}+{\left|\sum_{s=1}^{S}h_{u}w_{s}\right|}^{2}+{\left|h_{u}w_{g}\right|}^{2}+\sigma_{u}^{2}}$$

(17) $$\;s.t.\;{\sum_{u\in U}\left\|w_{u}\right\|}^{2}\leq P^{\left(c\right)},$$

where $P^{\left(c\right)}$ denotes the communication power and total transmitter power P consists of communication power $P^{\left(c\right)}$ and sensing power $P^{\left(s\right)}$, given by

(18) $$P=P^{\left(c\right)}+P^{\left(s\right)}.$$

Constraint (18) is a Pareto boundary constraint (power allocation trade-off). To determine the optimal solution for Problem (16), we establish a lower bound for the SINR applicable to all users.

(19) $${\mathrm{SINR}}_{u}=\gamma_{u},\;u=1,\ldots ,U,$$

(20) $$\gamma_{0}=\underset{\left\{u\right\}}{\min}\gamma_{u},$$

which implies ${\mathrm{SINR}}_{u}\geq \gamma_{0},\;\forall u\in U$. $\gamma_{0}$ denotes the lowest SINR received by *U* users. Thus, the constraint is given by

(21) $$\frac{{\left|h_{u}w_{u}\right|}^{2}}{\sum_{u\prime \in U/\{u\}}{\left|h_{u}w_{u\prime }\right|}^{2}+\sum_{s=1}^{S}{\left|h_{u}w_{s}\right|}^{2}+{\left|h_{u}w_{g}\right|}^{2}+\sigma_{u}^{2}}\geq \gamma_{0},$$

where we can re-write the constraint as

(22) $$(1+\frac{1}{\gamma_{0}}){\left|h_{u}w_{u}\right|}^{2}\geq {\left|h_{u}w_{u}\right|}^{2}+\sum_{u\prime \in U/\{u\}}{\left|h_{u}w_{u\prime }\right|}^{2}+\sum_{s=1}^{S}{\left|h_{u}w_{s}\right|}^{2}+{\left|h_{u}w_{g}\right|}^{2}+\sigma_{u}^{2}.$$

Due to N=U+S, it can be simplified to obtain

(23) $$(1+\frac{1}{\gamma_{0}}){\left|h_{u}w_{u}\right|}^{2}\geq \sum_{n=1}^{N}{\left|h_{u}w_{n}\right|}^{2}+{\left|h_{u}w_{g}\right|}^{2}+\sigma_{u}^{2}.$$

We take the square root of constraint (23) and reformulate it as a second-order cone constraint. Since the left-hand side of the inequality is a nonlinear function, we apply an arbitrary phase shift to transform the variable into its real part, a manipulation that preserves the squared norm, i.e.,

(24) huwu2=huwue−jψ=Re{huwu},

where ψ∈[0,2π] denotes the arbitrary angle. Constraint (23) can be expressed as

(25) (1+1γ0)Re{huwu}≥[huw1,…,huwN,huwg,σu]T.

Constraint (25) is formulated as a second-order cone constraint. Since the objective function is quasi-concave, the optimization problem in Problem (16) can be transformed into the following feasibility problem.

(26) $$\underset{\left\{w_{u}\right\}}{\max}\frac{{\left|h_{u}w_{u}\right|}^{2}}{{\left|\sum_{u\prime \in U/\{u\}}h_{u}w_{u\prime }\right|}^{2}+{\left|\sum_{s=1}^{S}h_{u}w_{s}\right|}^{2}+{\left|h_{u}w_{g}\right|}^{2}+\sigma_{u}^{2}}$$

(27) s.t. (1+1γ0)Re{huwu}≥[huw1,…,huwN,huwg,σu]T,

(28) $${\sum_{u\in U}\left\|w_{u}\right\|}^{2}\leq P^{\left(c\right)}.$$

In addressing Problem (26), the presence of unknown variables $\gamma_{0}$ and ${\left\{w_{s}\right\}}_{s\in S}$ makes it impractical to directly solve for ${\left\{w_{u}\right\}}_{u\in U}$. Recent studies have tackled this issue by introducing a lower bound as a surrogate for $\gamma_{0}$, treating $\gamma_{0}$ as a known parameter when optimizing the other variables [30]. However, the effectiveness of this approach depends critically on the choice of the fixed parameter; significant deviation from the true optimal value can degrade the performance of the remaining variables.

To ensure optimized communication performance for users and achieve a sufficiently high SINR, it is essential to determine ${\left\{w_{s}\right\}}_{s\in S}$, which in turn enhances the value of $\gamma_{0}$. Therefore, we adopt a communication-centric beamforming strategy to design ${\left\{w_{s}\right\}}_{s\in S}$ with the objective of maximizing $\gamma_{0}$. The sensing beamforming vectors, constructed via conjugate beamforming, are projected into the null space of the communication channels. This strategy effectively mitigates the interference of sensing transmissions on communication performance, thereby improving the user’s SINR. The variables ${\left\{{\bar{w}}_{s}\right\}}_{s\in S}$ and ${\left\{w_{s}^{\mathrm{NS}}\right\}}_{s\in S}$ can be expressed as follows:

(29) $${\bar{w}}_{s}^{\mathrm{NS}}=(I-H_{c}^{H}{\left(H_{c}H_{c}^{H}\right)}^{†}H_{c})a(\varphi_{s})$$

(30) $$w_{s}^{\mathrm{NS}}=\sqrt{P^{\left(s\right)}}\frac{{\bar{w}}_{s}^{\mathrm{NS}}}{\left\|{\bar{w}}_{s}^{\mathrm{NS}}\right\|}.$$

After the ${\left\{{\bar{w}}_{s}\right\}}_{s\in S}$ is replaced by the ${\left\{w_{s}\right\}}_{s\in S}$, constraint (25) can be reformulated as a second-order cone problem, which can be solved using a convex solver. Specifically, we apply the standard bisection method to determine the maximum SINR value, denoted as $\gamma_{0}$, that satisfies the given constraints. First, we establish $\gamma_{\min}$ and $\gamma_{\max}$ as the lower and upper bounds of the SINR range, respectively, initialized based on the theoretical limits of feasible SINR values. At each iteration, the algorithm computes the midpoint of the current interval and uses the CVX solver to assess its feasibility. If a feasible solution is obtained, the lower bound is updated to this midpoint; otherwise, the upper bound is reduced to it. This iterative process continues until the gap between the upper and lower bounds falls below or equals λ=0.01. By progressively narrowing the search interval, the method efficiently converges to an accurate approximation of the optimal SINR. Finally, we can determine the variable ${\left\{w_{u}\right\}}_{u\in U}$ in accordance with the optimal SINR.

Furthermore, a concise proof of convexity for problem (26) is required to establish a theoretical foundation for the convergence and optimality of the optimization algorithm. First, the power constraint ${\sum_{u\in U}\left\|w_{u}\right\|}^{2}\leq P^{\left(c\right)}$ defines a closed convex set, specifically a 2-norm ball in ${\mathbb{C}}^{N}$. Subsequently, objective function (27) can be reformulated as a constraint ${\mathrm{SINR}}_{u}\geq \zeta ,\;u=1,\ldots U$ featuring a quadratic function of ${\left\{w_{u}\right\}}_{u\in U}$ on the left-hand side and a constant on the right-hand side. Since $|h_{u}w_{u}{|}^{2}=w_{u}^{H}h_{u}^{H}h_{u}w_{u}$ is a semi-positive semi-definite quadratic form, the constraint inequality can be expressed as $w_{u}^{H}A_{k}w_{u}\geq b_{k}$, and its feasible domain is the convex set (the leader-layer set of the semi-positive definite quadratic function). Constrant (25) is transformed by constrant (23), and it also transforms the semi-positive definite quadratic form. The objective function is convex, and the feasible region defined by the constraints constitutes a convex set. Consequently, the leader-layer optimization problem qualifies as a convex optimization problem, and the bisection method is capable of converging to the global optimum under appropriate conditions. Additionally, the optimality of problem (26) should be verified by examining the corresponding dual analysis. Algorithm 1 provides a concise overview of the leader-layer optimization process. For detailed derivations and explanations, please refer to Appendix A.

In summary, this sub-section introduces a maximin optimization method that utilizes the sensing beamforming vector—constructed via conjugate beamforming—and the optimal communication beamforming vector as the final outputs. However, although the solution guarantees optimal communication performance, it does not necessarily achieve optimal sensing performance, as the strategy is inherently communication-centric. In the following section, we retain the optimized ${\left\{w_{u}\right\}}_{u\in U}$ derived in this section and use it as a fixed component to jointly optimize the sensing-centric beamforming vector ${\left\{w_{s}\right\}}_{s\in S}$. Furthermore, we derive the convergence of leader-layer optimization in Appendix B.1.
**Algorithm 1.** Leader-layer optimizationInput: $\gamma_{\min}$, $\gamma_{\max}$ and λOutput: ${\left\{w_{u}\right\}}_{u\in U}$      1Initialize:      2Convert (16) to ${\mathrm{SINR}}_{u}\geq \gamma_{0},\;u=1,\ldots U.$      3Convert (22) to second-order cone constraint (24).      4Compute ${\left\{w_{s}^{\mathrm{NS}}\right\}}_{s\in S}$ by (26) and (27).      5Replace ${\left\{w_{s}^{\mathrm{NS}}\right\}}_{s\in S}$ with ${\left\{w_{s}\right\}}_{s\in S}$.      6Maximize ${\mathrm{SINR}}_{u}$ with constraint (24) and $\sum_{u\in U}{\left\|w_{u}\right\|}^{2}\leq P^{\left(c\right)}$.      7Optimization of $\gamma_{0}$.      8Repeat      9         Establish $\gamma_{\min}(t)$ and $\gamma_{\max}(t)$ as the lower and upper bounds.      10         Compute the midpoint $\gamma_{0}(t)$ of the current range.      11         Use the CVX solvers to assess its feasibility.      12         If a feasible solution is obtained, $\gamma_{\min}(t+1)=\gamma_{0}(t)$.      13         Otherwise, $\gamma_{\max}(t+1)=\gamma_{0}(t)$.      14         Update midpoint $\gamma_{0}(t+1)$      15Until $\gamma_{\max}(t+1)-\gamma_{\min}(t+1)<\lambda$ *convergence*      16Obtain $\gamma_{0},\;{\left\{w_{s}^{\mathrm{NS}}\right\}}_{s\in S}$      17Solving for (25) results in ${\left\{w_{u}\right\}}_{u\in U}$.

### 3.2. Semi-Definite Programming Solvers Based on the CVX Toolbox

For the follower layer, the sensing performance is mainly measured based on the value of SCNR. Thus, we present a sensing SCNR maximization optimization problem, given by

(31) $$\underset{\left\{w_{s}\right\}}{\max}\;\mathrm{SCNR}$$

(32) $$s.t.{\sum_{s\in S}\left\|w_{s}\right\|}^{2}\leq P^{\left(s\right)}.$$

where the SCNR is defined in Equation (15).

We reformulated the SCNR optimization problem to an SDP which better optimizes the maximization problem, and $\alpha_{s}a(\phi_{s})a^{H}(\varphi_{s})W_{s}={\bar{G}}_{s},\;s=1,\ldots ,S$. Thus, the numerator of SCNR in Equation (15) can be simplified as

(33) $$\begin{array}{l} E[{\left\|\sum_{s=1}^{S}{\bar{G}}_{s}X_{s}\right\|}_{F}^{2}]=\sum_{s=1}^{S}E[{\left\|{\bar{G}}_{s}X_{s}\right\|}_{F}^{2}]\\ =\sum_{s=1}^{S}\mathrm{Tr}\left\{E[X_{s}X_{s}^{H}]\;E[{\bar{G}}_{s}^{H}{\bar{G}}_{s}]\right\}\\ =\sum_{s=1}^{S}\mathrm{Tr}\;E[{\bar{G}}_{s}^{H}{\bar{G}}_{s}], \end{array}$$

which is obtained by expansion of the Frobenius norm, interchanging expectation and trace, where $E[X_{s}X_{s}^{H}]=I$. Further, Equation (33) can be simplified as

(34) $$\begin{array}{l} =\sum_{s=1}^{S}\mathrm{Tr}\;E[{\left|\alpha_{s}\right|}^{2}a(\phi_{s})a^{H}(\varphi_{s})W_{s}W_{s}^{H}a(\varphi_{s})a^{H}(\phi_{s})]=\sum_{s=1}^{S}\zeta_{s}^{2}{\left\|a^{H}(\varphi_{s})W_{s}\right\|}^{2}\\ =\sum_{s=1}^{S}\zeta_{s}^{2}\mathrm{Tr}\;(A_{s}\sum_{s=1}^{S}{\bar{W}}_{s}), \end{array}$$

where $A_{s}=a(\phi_{s})a^{H}(\varphi_{s}),\;{\bar{W}}_{s}=w_{s}w_{s}^{H},\;\forall s\in S$, and the constraint ${\mathrm{SINR}}_{u}\geq \gamma_{0}$ are rewritten as

(35) $$(1+\frac{1}{\gamma_{0}})\mathrm{Tr}\;(Q_{u}{\bar{W}}_{u})-\mathrm{Tr}\;(Q_{u}\sum_{n=1}^{N}{\bar{W}}_{n})-\mathrm{Tr}\;(Q_{u}{\bar{W}}_{a})\geq \sigma_{u}^{2}$$

where $Q_{u}=h_{u}^{H}h_{u}$, ${\bar{W}}_{u}=w_{u}w_{u}^{H}$, and ${\bar{W}}_{g}=w_{g}w_{g}^{H}$.

In the following, we not only optimize the SCNR but also incorporate the revised SINR constraint Equation (36) into the SCNR optimization framework.

(36) $$\mathrm{Tr}\;(Q_{u}\sum_{s=1}^{S}{\bar{W}}_{s})\leq (1+\frac{1}{\gamma_{0}})\mathrm{Tr}\;(Q_{u}{\bar{W}}_{u})-\sigma_{u}^{2}-\mathrm{Tr}\;(Q_{u}\sum_{u=1}^{U}{\bar{W}}_{u})-\mathrm{Tr}\;(Q_{u}{\bar{W}}_{g}),$$

Thus, a reformulated SCNR optimization problem can be expressed as Problem (37).

(37) $$\underset{\left\{w_{s}\right\}}{\max}\sum_{s=1}^{S}\zeta_{s}^{2}\mathrm{Tr}\;(A_{s}\sum_{s=1}^{S}{\bar{W}}_{s})\;$$

(38) $$s.t.\;\mathrm{Tr}\;(Q_{u}\sum_{s=1}^{S}{\bar{W}}_{s})\leq (1+\frac{1}{\gamma_{0}})\mathrm{Tr}\;(Q_{u}{\bar{W}}_{u})-\sigma_{u}^{2}-\mathrm{Tr}\;(Q_{u}\sum_{u=1}^{U}{\bar{W}}_{u})-\mathrm{Tr}\;(Q_{u}{\bar{W}}_{g}),$$

(39) $$\sum_{s\in S}\mathrm{Tr}\;({\bar{W}}_{s})\leq P^{\left(s\right)},$$

where $Q_{u}$, ${\bar{W}}_{u}$, ${\bar{W}}_{g}$ and $\gamma_{0}$ are known. Problem (37) is an SDP that can be efficiently solved using CVX toolbox and standard convex SDP solvers [26,27]. The covariance matrix ${\bar{W}}_{s}$ obtained from the SDP solution typically has high rank (i.e., non-rank-1). Finally, we can obtain the sensing beamforming vector $w_{s}$.

Furthermore, a concise proof of convexity for problem (37) is required. The semi-positive definite constraint implies that $W_{s}≽0$ belongs to the cone of positive semi-definite matrices, which forms a convex cone. The power constraint on $\sum_{s\in S}\mathrm{Tr}\;({\bar{W}}_{s})\leq P^{\left(s\right)}$ is linear, and its feasible region thus constitutes a convex set. The objective function is linear, and the SDP problem is convex optimization subclass. Algorithm 2 provides a concise overview of the follower -layer optimization process.
**Algorithm 2.** Follower-layer optimization**Input:** ${\left\{h_{u},\;w_{u}\right\}}_{u\in U}$,
$w_{g}$ $\mathrm{and}\;\gamma_{0}$**Output:** ${\left\{w_{s}\right\}}_{s\in S}$      1**  Initialize:**      2  Convert numerator of SCNR to (30).      3  $\mathrm{Convert}\;{\mathrm{SINR}}_{u}\geq \gamma_{0}$ to (32).      4  $\mathrm{Compute}\;Q_{u}=h_{u}^{H}h_{u}$$,\;{\bar{W}}_{u}=w_{u}w_{u}^{H}$ $\mathrm{and}\;{\bar{W}}_{g}=w_{g}w_{g}^{H}$.      5  $\mathrm{Replace}{\left\{w_{s}^{\mathrm{NS}}\right\}}_{s\in S}$$\;\mathrm{with}{\left\{w_{s}\right\}}_{s\in S}$.      6  $\mathrm{Maximize}\;\sum_{s=1}^{S}\zeta_{s}^{2}\mathrm{Tr}\;(A_{s}\sum_{s=1}^{S}{\bar{W}}_{s})$ $\mathrm{with}\;\mathrm{constraint}\;(32)\;\mathrm{and}\sum_{s\in S}{\left\|w_{s}\right\|}^{2}\leq P^{\left(s\right)}$.      7  Convert problem to the SDP form.      8  $\mathrm{Optimization}\;\mathrm{of}{\left\{w_{s}\right\}}_{s\in S}$.      9  $\mathrm{Use}\;\mathrm{the}\;\mathrm{CVX}\;\mathrm{solvers}\;\mathrm{to}\;\mathrm{assess}\;\mathrm{its}\;\mathrm{feasibility}\;({\bar{W}}_{s}$ is non-rank-1).      10  $\mathrm{Solving}\;\mathrm{for}\;(33)\;\mathrm{results}\;\mathrm{in}{\left\{w_{s}\right\}}_{s\in S}$.


This hierarchical optimization solution prioritizes communication performance at the leader-layer level, thereby implementing a communication-centric design. However, while optimizing the communication beamforming vectors, the solution simultaneously accounts for sensing performance. Consequently, the approach not only significantly enhances communication quality but also achieves near-optimal performance for both sensing and communication functionalities. Furthermore, we derive the convergence of follower-layer optimization in Appendix B.2.

To assess whether the hierarchical optimization outcome approaches global optimality, we employ the sensing-and-communication mutual information (S&C MI) rate as a unified metric for evaluating integrated S&C performance. The S&C MI rate is defined as

(40) $$R_{sc}=\omega_{c}R_{c}+\omega_{s}R_{s},$$

(41) $$R_{c}=\log\det(I+\sum_{u\in U}{\mathrm{SINR}}_{u}),$$

(42) $$R_{s}=\log\det(I+\mathrm{SNR}).$$

where $\omega_{c}$ denotes the communication power ratio, and $\omega_{s}$ denotes the sensing power ratio, $\omega_{c}+\omega_{s}=1$. The S&C MI combines sensing mutual information (SMI) and communication mutual information (CMI) as a novel metric for assessing the performance of ISAC. The CMI rate represents the maximum achievable channel coding rate, while SMI is an increasing function of SNR, and both metrics exhibit similarities in their physical and mathematical properties. In addition, their mathematical isomorphism is evident in shared concave–convex properties and logarithmic dependencies—enabling direct cross-domain performance comparison while preserving the unique physical interpretations of each subsystem.

In the subsequent analysis, it is necessary to evaluate the trade-off between communication and sensing performance based on the S&C MI rate. We minimize sensing interference on communication by projecting the sensing beam ($w_{s}$) onto the null space of the communication channel ($h_{u}$) as formalized in Equation (26), thereby reducing the ${\left|\sum_{s=1}^{S}h_{u}w_{s}\right|}^{2}$ term in the denominator of Equation (10). The underlying principle of this optimization framework is to shrink the inferior region of the Pareto frontier, effectively driving the SINR value closer to their theoretical upper bounds under a given power budget. This results in a more compact and efficient Pareto boundary, which is subsequently validated through simulation. Notably, SINR–SCNR coupling can influence the Pareto frontier, and we provide analytical expressions in Appendix C.

In the following, we analyze the complexity of the algorithm; the computational complexity of ISAC hierarchical optimization communication to sensing (ISAC-OCS) consists of two parts, where the complexity of solving the bisection method is $𝒪{((\log}_{2}Y)U(UL^{3}N_{t}^{3}))$ and the complexity of solving the SDP problem is $𝒪(L^{3.5}N_{t}^{3.5}(US))$. Thus, the complexity of ISAC-OCS is $𝒪{((\log}_{2}Y)(U^{2}L^{3}N_{t}^{3})+L^{3.5}N_{t}^{3.5}(US))$ and the complexity of maximize–minimize optimization (MMO) is $𝒪{((\log}_{2}Y)(U^{2}L^{3}N_{t}^{3}))$ in [26]. We compare the complexity of other algorithms; the complexity of Weighted Minimum Mean Square Error ISAC (WMMSE-ISAC) is $𝒪((U+1)N_{t}^{3}+UR^{3}+N_{r}^{3})$ in [25] and the complexity of CM waveform design is $𝒪(L^{3}N_{R}^{3}+N_{D}N_{A}(2M+3)L^{2}N_{T}^{2})$ in [29].

## 4. Simulation Results

In this section, to verify the effectiveness of ISAC-OCS, we evaluate the sensing and communication performance associated with the previously discussed ISAC beamforming solutions. We consider a scenario in which the transmit RSU simultaneously delivers U=5 data streams to UEs and transmits S=1 sensing signal to the target, while the receive RSU captures C=3 clutter patches. Both the transmit RSU and BS are each equipped with $N_{t}=16$ transmit antennas, and the receive RSU is additionally equipped with $N_{r}=16$ receive antennas. The parameters for the communication model include a specified communication noise variance $\sigma_{u}^{2}=1$. For the sensing model, we consider parameters such as sensing noise variance $\sigma_{s}^{2}=1$ and sensing channel gain variance $\zeta_{s}=\zeta_{c}=1$. Additionally, we set the transmit power of the ISAC transmitter at *P* = 0 dBW, along with a defined Lagrangian parameter λ=0.1 and noise variance $\sigma^{2}=1$. In our analysis, we randomize the positions of the transmit RSU, receive RSU, UEs, and target, resulting in randomized azimuth angles for these entities. Under this setup, we conduct a comparative evaluation of the MMO beamforming method [26] and the WMMSE-ISAC method [25] in relation to the ISAC-OCS. Notably, ISAC-OCS demonstrates a significant advancement in methodological design compared to MMO and WMMSE-ISAC. MMO focuses solely on communication beamforming design and lacks an integrated perceptual SCNR optimization component. Although WMMSE-ISAC combines perceptual mutual information and communication mutual information through weighted summation into a unified objective for joint optimization, this approach is susceptible to substantial performance degradation in one of the functionalities due to inappropriate weight selection. All simulation experiments presented in this section were conducted in a standardized computational environment to ensure the reliability and reproducibility of the results. For the software framework, MATLAB R2024a was employed as the core platform. In terms of hardware configuration, the experiments were run on a workstation equipped with an Intel® Core™ i9-14900HX processor.

**Figure 2:**
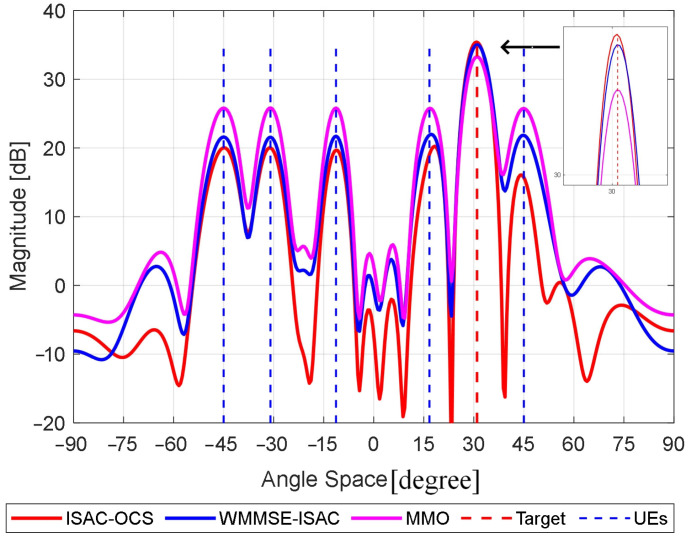
Multi-beam pattern comparisons for three methods.

To evaluate the angular resolution of various beamforming techniques, we conduct a comparative analysis of the beam patterns generated by three distinct methods, with the communication power ratio set to 0.5. As illustrated in Figure 2, the proposed multi-beam architecture simultaneously incorporates both communication-directed and sensing-oriented beams, necessitating joint evaluation using two critical metrics, (i) the absolute peak intensity of beam lobes, and (ii) inter-peak isolation, characterized by null depth in concave regions.

The MMO method adopts a communication-centric beamforming strategy, resulting in a beam pattern with lower peaks in the sensing beams (approximately 2.3 dB lower than ISAC-OCS) and higher peaks in the communication beams (up to 6.2 dB higher than ISAC-OCS) compared to the other approaches. However, its shallow inter-beam null depths fundamentally limit angular discrimination capability due to insufficient suppression of sidelobe interference. In contrast, the ISAC-OCS solution enhances the peak gains of sensing beams (peak gain > 0.9 dB relative to WMMSE-ISAC) at the expense of communication beam attenuation (peak reduction ≈ 1.8 dB relative to WMMSE-ISAC). Notably, it achieves significantly deeper null depths (≥12 dB) between adjacent beams compared to both MMO and WMMSE-ISAC, owing to stronger interference suppression. By analyzing the communication beam peak characteristics of this solution, we conclude—as previously discussed—that it does not follow a conventional communication-centric beamforming strategy. Instead, it enables joint optimization of communication and sensing performance. In summary, although the ISAC-OCS yields relatively lower communication peaks, it exhibits more pronounced concave drops between adjacent lobes, thereby enhancing beamforming angular resolution.

**Figure 3:**
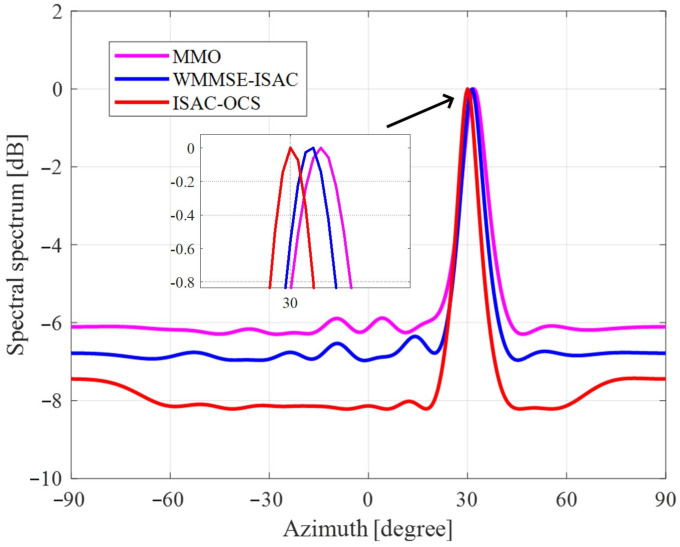
Normalized spectrum of the MUSIC algorithm for three beamforming methods.

After clutter suppression is applied to the received echo signals, target angle estimation is performed. Figure 3 shows the normalized spatial spectrum obtained via the MUSIC algorithm for three optimized beamforming methods. As a high-resolution subspace method, the MUSIC algorithm theoretically achieves superior resolution compared to conventional beamforming techniques, exceeding the limitations imposed by the Rayleigh criterion. Given that the experiment focuses on angle estimation for a single target, the utilization of four receiving antenna sensors suffices. The findings demonstrate that three optimized methods effectively detect the target angle; however, the beam spectrum peak generated by the ISAC-OCS design is notably the sharpest, signifying enhanced spatial resolution and more effective sidelobe suppression. The collateral valve inhibition achieved by ISAC-OCS exceeds that of MMO by more than 1.6 dB and outperforms WMMSE-ISAC by over 1.2 dB. Furthermore, the angle estimation accuracy associated with ISAC-OCS surpasses that of the other two methods, thereby corroborating its superior sensing performance.

**Figure 4:**
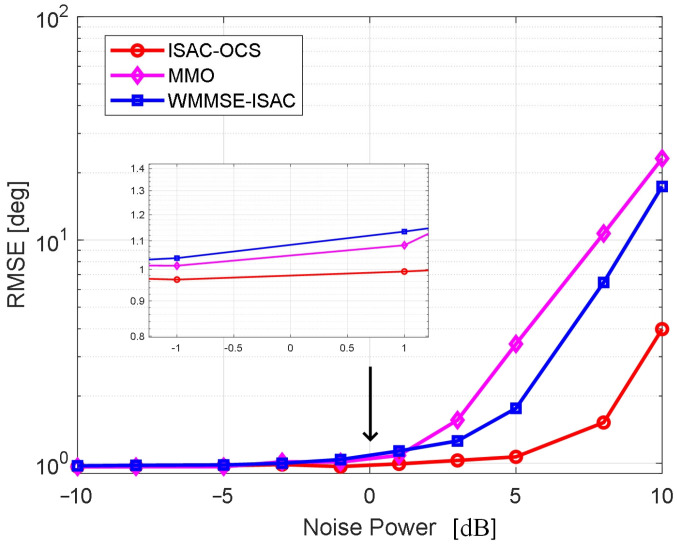
RMSEs versus the noise power for three beamforming methods.

The Root Mean Square Error (RMSE) obtained from DOA estimation serves as a crucial metric for evaluating the perceptual performance of beamforming design. The experimental framework employs rigorous Monte Carlo validation to ensure a comprehensive performance assessment. Figure 4 illustrates a comparison of the RMSE in angle estimation across the three optimized beamforming methods’ varying noise power levels. As noise power diminishes to lower values, the RMSE values for all three methods converge, indicating minimal differences in estimation performance under low-noise conditions. Nevertheless, throughout the entire noise power spectrum, the RMSE corresponding to ISAC-OCS remains consistently lower than other beamforming methods, evidencing its ability to sustain high estimation accuracy even in complex noise environments. This result further substantiates the reliability of the ISAC-OCS beamforming design approach with respect to sensing performance.

**Figure 5:**
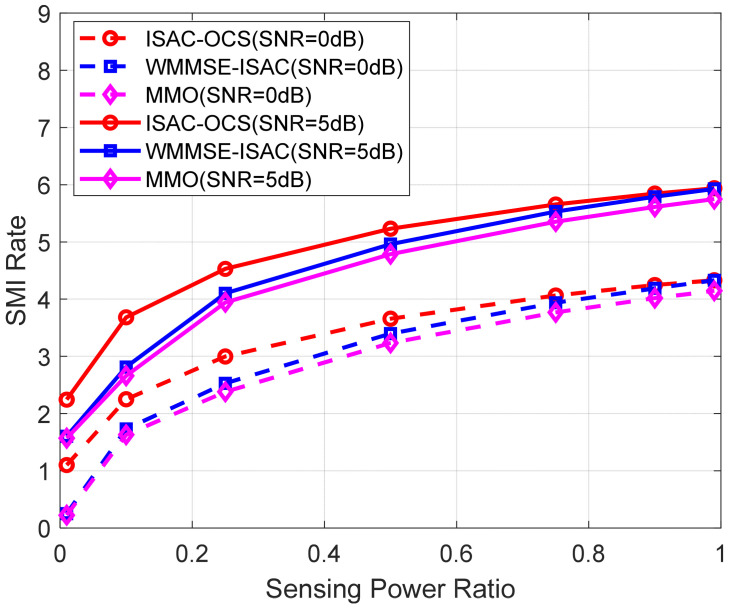
The SMI rate versus sensing power ratio for three beamforming methods.

Beyond angular resolution, the joint performance of communication and sensing is evaluated via SMI and CMI. With the sensing power ratio ranging from 0.01 to 0.99, the SMI rate at SNR = 0 dB and SNR = 5 dB is illustrated in Figure 5. This investigation evaluates the characteristics of SMI across three distinct methodologies under varying conditions of sensing power allocation and signal quality.

The systematic comparison demonstrates that the ISAC-OCS technique consistently outperforms both WMMSE-ISAC and MMO methods in terms of sensing performance, with this advantage remaining evident across various channel quality conditions. All methods examined exhibit fundamentally similar behavioral patterns, where enhanced signal conditions yield proportional improvements in SMI rates at fixed sensing power configurations. When the sensing power is 0.2, the SMI rate of ISAC-OCS is about 0.65 dB higher than that of MMO and approximately 0.1 dB higher than that of WMMSE-ISAC. The overall findings indicate that although all approaches experience improvements in enhanced signal environments, the ISAC-OCS method emerges as the most robust solution for improving sensing performance.

**Figure 6:**
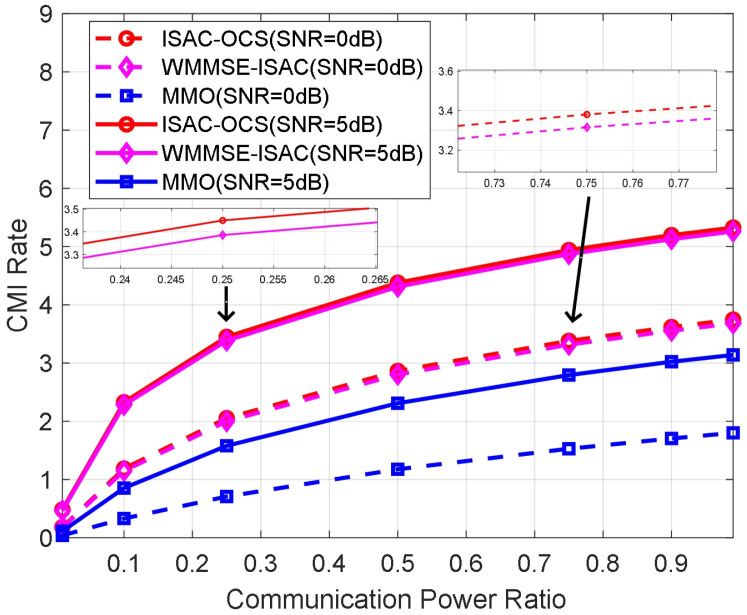
The CMI rate versus communication power ratio for three beamforming methods.

For communication performance, Figure 6 considers two scenarios in which the transmitting SNR is established at 0 dB and 5 dB, respectively. The figure illustrates the fluctuation of the CMI rate in relation to the communication power ratio, utilizing three distinct beamforming optimization solutions across different SNR levels. For each curve, 200 Monte Carlo experiments are performed. In Figure 6, all methodologies exhibit fundamentally similar behavioral patterns, characterized by an enhancement in CMI rates as the allocation of communication power increases across the entire operational range. Notably, the MMO and ISAC-OCS techniques, which share identical theoretical frameworks for deriving beamforming vectors, demonstrate almost indistinguishable communication performance results (the CMI rate of ISAC-OCS is marginally higher than that of MMO, with a difference of approximately 0.08 dB). Both methodologies consistently outperform the WMMSE-ISAC approach across all assessed SNR conditions, thereby confirming their superior efficacy in maintaining communication quality.

**Figure 7:**
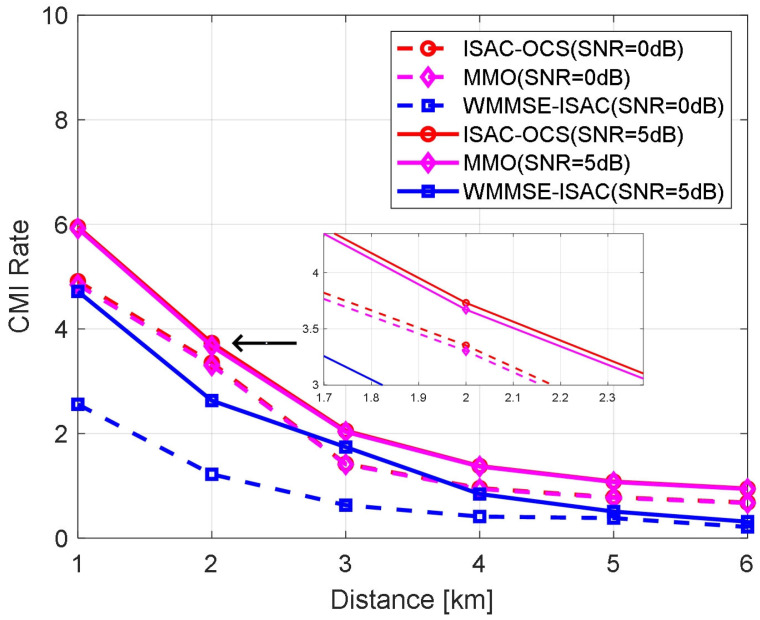
The CMI rate versus UE distance for three beamforming methods.

Figure 7 examines the CMI rate of the three optimized beamforming methods as a function of user distance under varying transmission SNRs. The experimental results reveal that, irrespective of transmission SNR fluctuations, the CMI rates for ISAC-OCS and MMO outperform those of WMMSE-ISAC. Although ISAC-OCS and MMO exhibit comparable overall trends, a detailed analysis indicates that ISAC-OCS achieves marginally higher rates (the CMI rate of ISAC-OCS is marginally higher than that of MMO, with a difference of approximately 0.09 dB), highlighting its advantage in communication performance.

**Figure 8:**
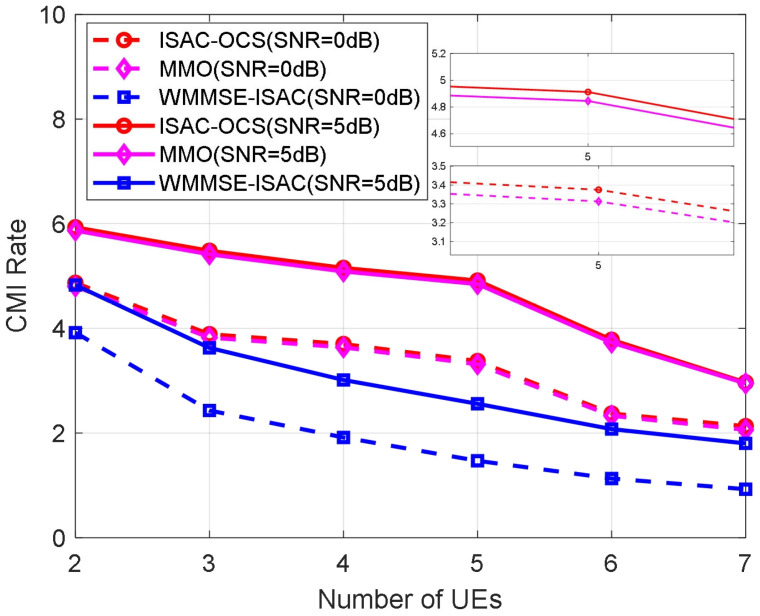
The CMI rate versus number of UEs for three beamforming methods.

Similarly, Figure 8 evaluates the CMI rate as the number of users varies under different transmission SNR conditions, yielding results consistent with those in Figure 6 and Figure 7: the CMI rate for ISAC-OCS consistently exceeds that of MMO and is substantially superior to WMMSE-ISAC (the CMI rate of ISAC-OCS is marginally higher than that of MMO, with a difference of approximately 0.07 dB). These findings collectively affirm the comprehensive performance benefits of the ISAC-OCS beamforming strategy within integrated communication and sensing systems. The experimental results substantiate that the proposed methodology achieves communication performance comparable to that of MMO while substantially outperforming WMMSE-ISAC. This finding highlights the necessity for further investigation into the comparative sensing performance characteristics of these competing schemes, as the communication-sensing trade-off is a critical design consideration in integrated systems.

**Figure 9:**
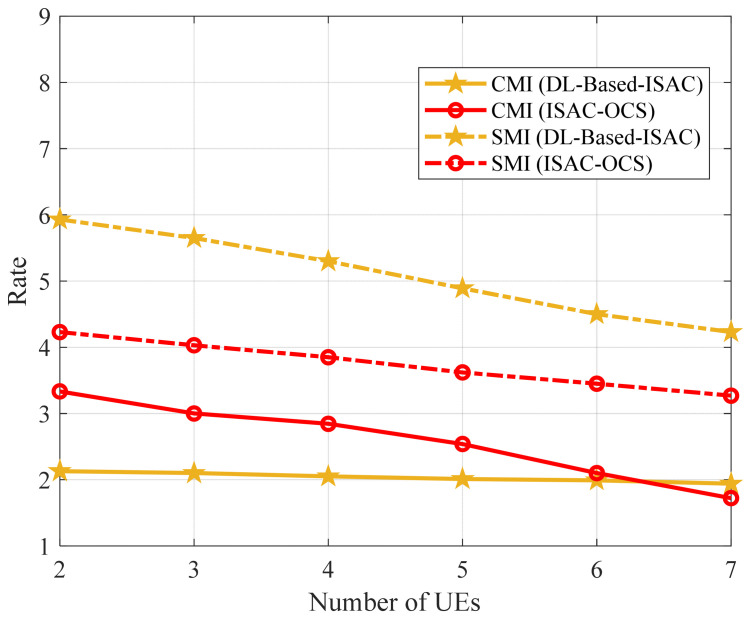
The performance versus the number of UEs for two methods.

Furthermore, it is observed that the CMI rate decreases as the number of UEs increases. This is because more UEs means more communication interference. On the one hand, more MUI degrades the performance of communication. On the other hand, the more communication to sensing interference leads to the decrease in SMI rate. In Figure 9, we compare the ISAC-OCS hierarchical optimization method with the deep learning-based method [31], observing the differences in the CMI rate and SMI rate between the two methods.

As the number of UEs increases, the rate of all four schemes exhibits a decreasing trend. The SMI (DL-based ISAC) consistently achieves a higher rate than SMI (ISAC-OCS) across all UE numbers. As the number of UEs increases from 2 to 6, the CMI (ISAC-OCS) achieves a higher rate than CMI (DL-based ISAC). The advantage of DL-based ISAC may stem from its advanced deep learning-driven resource allocation and information processing mechanisms, which enable better adaptability to the growing number of UEs. However, the DL-based ISAC method is highly reliant on high-quality training data. Performance degradation occurs significantly when there is a discrepancy between the actual operational scenario and the training dataset. In contrast, ISAC-OCS does not require an offline training phase. Although its SMI rate is lower than that of DL-based approaches, it operates without dependence on specialized computing hardware such as GPUs. Moreover, unlike DL-based ISAC methods, which incur substantial computational costs during model training, ISAC-OCS achieves efficient resource utilization. Consequently, ISAC-OCS demonstrates superior adaptability in complex and dynamic environments, particularly with respect to computational efficiency and practical deployment.

While ISAC-OCS demonstrates communication performance comparable to MMO, it has been specifically optimized for sensing capabilities, leading to enhanced sensing performance relative to the MMO method and superior overall performance compared to WMMSE-ISAC, as supported by Figure 3, Figure 4 and Figure 5. To further explore the joint communication and sensing performance across different methods, we analyze the S&C rate in relation to power allocation and SNR.

**Figure 10:**
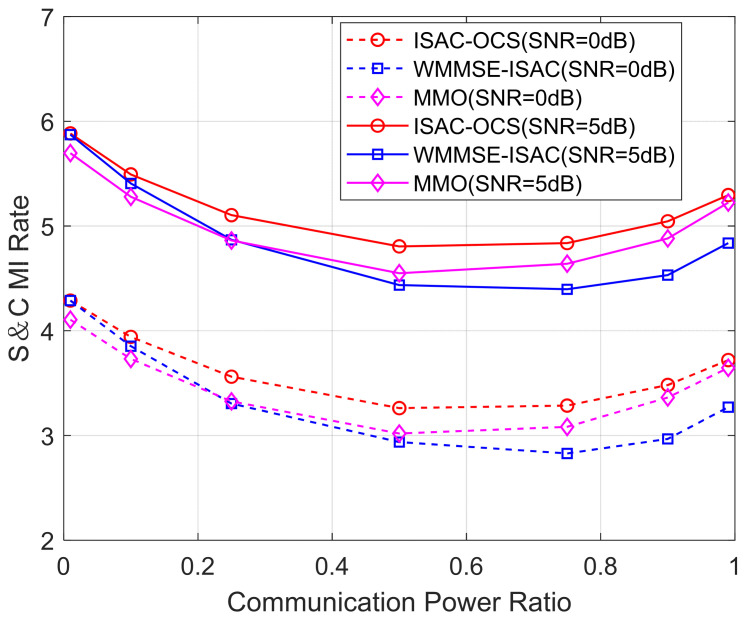
The S&C rate versus communication power allocation for three beamforming methods.

In Figure 10, we investigate S&C rate variations as functions of communication power ratios for different schemes under transmit SNR values of 0 dB and 5 dB, respectively. Each performance curve is obtained by averaging over 200 independent Monte Carlo trials to ensure statistical robustness. As anticipated, the S&C rate curves corresponding to higher SNRs consistently surpass those obtained at lower SNRs, indicating the significant influence of signal quality on overall system performance. Among the evaluated methods, ISAC-OCS exhibits marginally higher S&C rates than both WMMSE-ISAC and MMO across the entire range of communication power ratios (when the communication power is approximately 0.5, the S&C MI rate of ISAC-OCS is about 0.41 dB higher than that of MMO and approximately 0.29 dB higher than that of WMMSE-ISAC).

Furthermore, it is observed that the S&C rates achieved at intermediate power allocation settings ($\omega_{c}=0.5$ and $\omega_{c}=0.75$) are generally lower than those obtained under extreme optimization biases ($\omega_{c}=0.01$ or $\omega_{c}=0.99$). This indicates that the overall performance at these intermediate points fails to match the specialized effectiveness achieved in purely sensing-centric or communication-centric configurations. However, allocating power according to balanced ratios ($\omega_{c}=0.5$ and $\omega_{c}=0.75$) is beneficial in scenarios that require the simultaneous fulfillment of both sensing and communication needs, providing a trade-off solution under practical system constraints. In contrast, the adoption of extreme power allocation ($\omega_{c}=0.01$ or $\omega_{c}=0.99$) is only appropriate for scenarios dominated by either communication or sensing demands and may result in significant performance degradation if applied indiscriminately in integrated operational environments.

**Figure 11:**
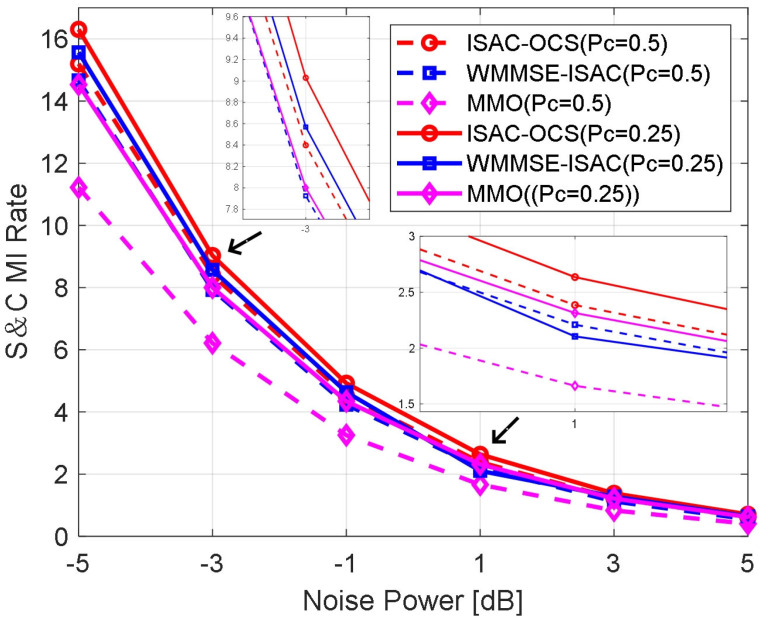
The S&C rate versus noise power for three beamforming methods.

In Figure 11, the communication power ratios are set at 0.5 and 0.25, respectively. The figure illustrates the variation in the S&C MI rate curves as functions of SNR for the different examined methods. Evidently, the S&C MI rates achieved by the ISAC-OCS scheme consistently surpass those attained by the baseline approaches across the entire SNR range (when the noise power is approximately 1, the S&C MI rate of ISAC-OCS is about 0.52 dB higher than that of MMO and approximately 0.28 dB higher than that of WMMSE-ISAC). This enhanced performance highlights the efficacy of ISAC-OCS in simultaneously optimizing sensing and communication objectives.

**Figure 12:**
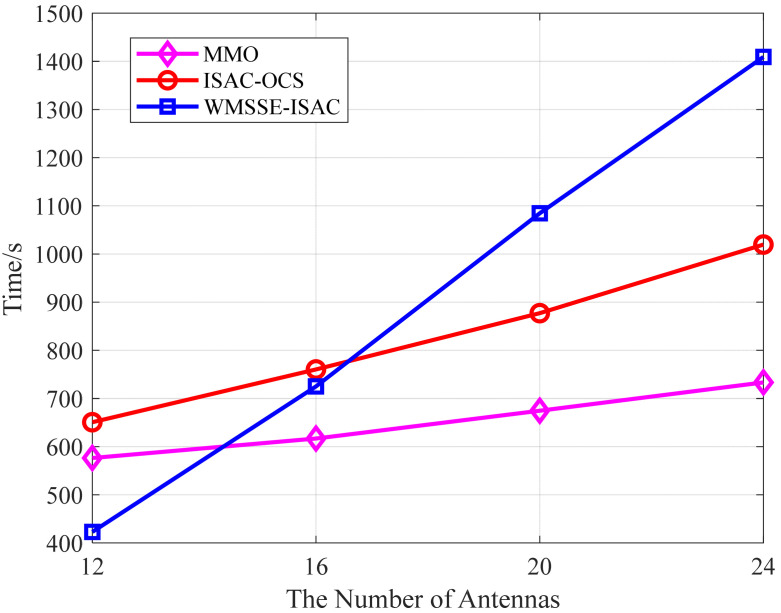
Variation in the hundred run times with the number of antennas.

At the end of the preceding section, we analyze the asymptotic complexity of the three methods. The ISAC-OCS method is distinguished by its combination of the bisection method and convex optimization, which results in a notably higher complexity order compared to MMO. Figure 12 illustrates the runtime curves of the three methods as a function of the number of antennas.

As illustrated in Figure 12, MMO exhibits the shortest runtime across various specification arrays, while ISAC-OCS exhibits a longer runtime compared to MMO. Notably, WMMSE-ISAC incurs a greater computational time than ISAC-OCS as the number of array elements increases ($N_{t}=N_{r}=20$ and $N_{t}=N_{r}=24$). Therefore, MMO and WMMSE-ISAC exhibit the lowest and highest levels of complexity compared to the others, aligning with the previous analysis.

In summary, the CMI rate, SMI rate, and S&C MI rate provided by ISAC-OCS are among the fastest compared to the other methods. Although the computational complexity of ISAC-OCS is slightly higher compared to the MMO, it achieves a superior balance between communication and sensing performance while maintaining a high S&C MI rate. Consequently, the moderate increase in computational complexity is justifiable for practical implementation in real-world ISAC-RSU systems.

## 5. Conclusions

In this paper, we propose a hierarchical optimization solution for ISAC beamforming, specifically tailored for ISAC-RSU systems operating in complex environments. We decompose the complex joint optimization problem into multiple interrelated sub-optimization layers. On the one hand, communication beamforming was treated as leader-layer optimization. The proposed model initially employed a maximize–minimize optimization strategy to achieve SINR optimization and utilized the bisection method to determine the maximum SINR value, ultimately solving the communication beamforming vector. On the other hand, the sensing beamforming functioned as follower-layer optimization. We employ SDP solvers based on the CVX toolbox to solve the sensing SCNR maximization problem, thereby facilitating the collaborative resolution of the global objective. This optimized solution not only enhances communication performance for receiving users but also significantly improves the sensing capabilities of the ISAC-RSU systems. Despite the increased complexity of the method, simulation results indicate that the proposed model exhibits superior sensing and communication performance compared to the others. The results presented above further underscore the efficacy of ISAC-OCS in achieving an optimal balance between communication and sensing, effectively managing the inherent trade-offs characteristic of ISAC systems. In summary, these results demonstrate the potential of ISAC-OCS as a comprehensive solution for next-generation RUS systems that demand flexible management of trade-offs between communication throughput and sensing accuracy. Looking ahead, we intend to investigate a sophisticated coordination mechanism capable of dynamically allocating resources based on real-time demands and environmental conditions, thereby ensuring that neither communication nor sensing performance is unduly compromised. This mechanism is expected to further validate the effectiveness of ISAC-OCS in harmonizing the dual objectives of ISAC systems, offering a promising framework for future ISAC networks where both capabilities must coexist seamlessly. In addition, our next step will involve initiating research on dynamic channels and channel estimation errors. Specifically, we will aim to utilize sensing-aided echo signals to correct channel estimation inaccuracies, thereby improving system robustness. We will incorporate the time-varying nature of clutter in urban environments and will exploit the real-time sensing capability of the ISAC-RSU proposed in this paper to ensure that SCNR optimization remains consistently aligned with the actual surrounding conditions. Subsequently, we will introduce a “Doppler frequency offset estimation module” to address mobile targets or UEs. This module will enable real-time updates of UE channels, allowing for dynamic adjustment of both the communication and the sensing beamforming vector. In our future work, we also plan to explore adaptive environment-aware predictive beamforming using DL-based techniques to enhance the real-time adaptability and overall efficiency of integrated sensing and communication systems. Such advancements will support emerging applications such as autonomous driving, intelligent infrastructure monitoring, and real-time human–machine interaction.

## Data Availability

The data presented in this study are available on request from the corresponding author. the data are not publicly available due to privacy restrictions.

## Abbreviations

The following abbreviations are used in this manuscript:

ISAC	Integrated Sensing and Communication
RF	Radio Frequency
RSU	Roadside Unit
V2X	Vehicle-to-Everything
SLAM	Simultaneous Localization and Mapping
BS	Base Station
UE	User Equipment
SCNR	Signal-to-Clutter-Plus-Noise Ratio
SINR	Signal-to-Interference-plus-Noise Ratio
SNR	Signal-to-Noise Ratio
SDP	Semi-Definite Programming
MIMO	Multiple Input Multiple Output
WMMSE	Weighted Minimum Mean Square error
MI	Mutual Information
MAI	Multiple Access Interference
MUI	Multi-User Interference
RCS	Radar Cross-Section
S&C	Sensing and Communication
ULA	Uniform Linear Array
N-LOS	Non-Line-of-Sight
DOA	Direction of Arrival
DOD	Direction of Departure
MMO	Maximize-Minimize Optimization

## Appendix A

For dual analysis, we first construct the Lagrange function

(A1) $$ℒ(\{w_{u}\},\gamma_{0},\mu_{0},\{\nu_{u}\})=\gamma_{0}-\mu_{0}(\sum_{u}∥w_{u}{∥}^{2}-P^{\left(c\right)})-\sum_{u}\nu_{u}(\gamma_{0}-\frac{{\left|h_{u}w_{u}\right|}^{2}}{\sum_{u\prime \in U/\{u\}}{\left|h_{u}w_{u\prime }\right|}^{2}+\sum_{s=1}^{S}{\left|h_{u}w_{s}\right|}^{2}+{\left|h_{u}w_{g}\right|}^{2}+\sigma_{u}^{2}}),$$

where μ≥0 denotes the dual variable of power constraint, $\nu_{u}\geq 0$ denotes the SINR constraint dual variable of the *u*-th UE, and $\sum_{u}\nu_{u}=1$ denotes normalization conditions for the dual problem.

The dual function is defined as $d(\mu ,\{\nu_{u}\})=\max_{\{w_{u}\},\gamma_{0}}ℒ$, and the dual problem can be expressed as

(A2) $$\underset{\mu \geq 0,\nu_{u}\geq 0}{\min}d(\mu ,\{\nu_{u}\})$$

(A3) $$s.t.\sum_{u}\nu_{u}=1$$

Subsequently, we obtain the feasible point $\{w_{u}^{0}\}=\epsilon \cdot \mathrm{argmax}\;\underset{u}{\min}{\left|h_{u}w\right|}^{2}$ (ϵ>0), and it strictly meets the power constraint $\sum_{u}∥w_{u}{∥}^{2}=\epsilon^{2}<P^{\left(c\right)}$. Therefore, the SINR of the feasible point can be expressed as

(A4) $${\mathrm{SINR}}_{u}(\{w_{u}^{0}\})=\frac{\epsilon^{2}{\left|h_{u}w_{u}^{0}\right|}^{2}}{\epsilon^{2}\sum_{u\prime \in U/\{u\}}{\left|h_{u}w_{u\prime }^{0}\right|}^{2}+\sum_{s=1}^{S}{\left|h_{u}w_{s}\right|}^{2}+{\left|h_{u}w_{g}\right|}^{2}+\sigma_{u}^{2}}$$

Finally, since ${\mathrm{SINR}}_{u}(\{w_{u}^{0}\})>\gamma_{\min}$ strictly meets the SINR constraint, $\gamma_{\min}$ is the minimum threshold for the value of γ. Therefore, the Slater condition is satisfied, implying that strong duality holds and the optimal value of the primal problem is equal to that of the dual problem.

## Appendix B

### Appendix B.1

We prove that the convergence of leader-layer optimization, which combines the max–min SINR approach with the bisection method, is rigorously established. The detailed proof is presented as follows:

The core logic of the leader layer, which employs the “binary search method for solving max-min SINR,” is to iteratively narrow the feasible region of SINR, defined as [$\gamma_{\min}$, $\gamma_{\max}$], in order to determine the maximum achievable $\gamma_{0}$ that satisfies the given power constraint.

(i) Monotonicity

In each iteration, if the midpoint $\gamma_{\mathrm{mid}}=(\gamma_{\min}+\gamma_{\max})/2$ is feasible (i.e., there exists a vector $w_{u}$ that satisfies ${\mathrm{SINR}}_{u}\geq \gamma_{\mathrm{mid}}$), then update $\gamma_{\min}=\gamma_{\mathrm{mid}}$; otherwise, update $\gamma_{\max}=\gamma_{\mathrm{mid}}$. The length of the feasible region, given by $\Delta \gamma =\gamma_{\max}-\gamma_{\min}$, decreases exponentially with respect to the number of iterations *k*: $\Delta \gamma^{\left(k\right)}=\Delta \gamma^{\left(0\right)}/2^{k}$.

(ii) Convergence

When the termination condition Δγ≤λ (where λ = 0.01 in this paper) is satisfied, $\gamma_{\mathrm{mid}}$ converges to the true optimal value $\gamma_{0}$, and the convergence rate is $𝒪(1/2^{k})$, indicating exponential convergence. At this point, the corresponding communication beamforming vectors $w_{u}$ can be obtained by solving a second-order cone program using the CVX toolbox. The solution is unique, as the second-order cone constraints define a strictly convex feasible set and the objective function is quasi-concave, ensuring the existence and uniqueness of the optimal solution.

### Appendix B.2

We prove the convergence of follower-layer optimization, and follower layer reformulates the SCNR maximization problem as an SDP, with convergence ensured by the strong duality property inherent in convex optimization.

The objective function of follower-layer optimization can be reformulated as a linear function with respect to ${\bar{W}}_{s}=w_{s}w_{s}^{H},\;\forall s\in S$, while the constraint is expressed as a linear matrix inequality (LMI). This formulation results in a strictly convex optimization problem.

According to the strong duality theory in convex optimization, the optimal value of the primal SDP problem is equal to that of its dual problem, and there exists an optimal solution $W_{s}^{*}$. When solving this problem using the CVX toolbox via an interior-point method, the algorithm converges to the global optimum, with convergence accuracy guaranteed by the default solver settings, such as a duality gap tolerance of no more than $10^{-8}$.

In summary, leader-layer optimization converges uniquely to $w_{u}^{*}$, and with $w_{u}^{*}$ fixed, follower-layer optimization converges uniquely to $w_{s}^{*}$. Therefore, the entire hierarchical optimization scheme achieves convergence within a finite number of iterations. Specifically, only one binary search iteration at the upper level (to attain $w_{u}^{*}$) and one SDP solution at the lower level (to attain $w_{s}^{*}$) are required to obtain the globally optimal solution, eliminating the need for iterative cycling between levels.

## Appendix C

We provide analytical expressions showing how SINR–SCNR coupling influences the Pareto frontier through three systematic steps: quantification of coupling terms, derivation of modified performance metrics, and analysis of the conditions defining the Pareto frontier. The detailed derivation is provided as follows:

(i) Quantification of coupling terms

The fundamental coupling mechanism between SINR and SCNR stems from the contribution of communication signals to sensing noise, as well as the contribution of sensing signals to communication interference. It is essential to first explicitly characterize this coupling effect.

First, we define the sensing–communication interference coupling coefficient α as a metric that quantifies the extent of interference imposed by the sensing beam on the communication link. This coefficient is determined by the degree of overlap between the sensing beam vector $w_{s}$ and the communication channel vector $h_{u}$.

(A5) $$\alpha =\frac{E\left[{\left|\sum_{s=1}^{S}h_{u}w_{s}x_{s}[l]\right|}^{2}\right]}{E\left[{\left|h_{u}w_{u}x_{u}[l]\right|}^{2}\right]}=\frac{\sum_{s=1}^{S}{\left|h_{u}w_{s}\right|}^{2}}{{\left|h_{u}w_{u}\right|}^{2}},$$

where the numerator denotes the interference power imposed by the sensing signal on the *u*-th UE, while the denominator represents the expected communication signal power received by the UE. A value of α>0 indicates the presence of coupling, whereas α=0 signifies the absence of coupling.

Subsequently, we define the communication–sensing interference coupling coefficient β as a metric that quantifies the proportion of clutter introduced by the communication beam into the sensing link. This coefficient is determined by the degree of overlap between the communication beamforming vector $w_{u}$ and the sensing channel matrix $G_{s}$.

(A6) $$\beta =\frac{E\left[{\left|\sum_{u=1}^{U}h_{u}w_{u}x_{u}[l]\right|}^{2}\right]}{E\left[{\left|\sum_{s=1}^{S}G_{s}w_{s}x_{s}[l]\right|}^{2}\right]}=\frac{\sum_{u=1}^{U}\zeta_{c}^{2}{\left|a^{H}(\theta_{u})w_{u}\right|}^{2}}{\sum_{s=1}^{S}\zeta_{s}^{2}{\left|a^{H}(\varphi_{s})w_{s}\right|}^{2}}$$

where the numerator denotes the interference power of the communication signal at the sensing receiver, while the denominator represents the expected power of the sensing echo signal. A value of β>0 indicates the presence of coupling, whereas β=0 signifies the absence of coupling.

(ii) Derivation of modified performance metrics

We integrate the ${\mathrm{SINR}}_{u}$ and SCNR equations and substitute the coupling coefficients α and β into these expressions; we derive the modified performance metrics under the influence of coupling effects. Specifically, we substitute $\sum_{s=1}^{S}{\left|h_{u}w_{s}\right|}^{2}=\alpha {\left|h_{u}w_{u}\right|}^{2}$ into the ${\mathrm{SINR}}_{u}$ expression and set the communication MUI to $I_{c}=\sum_{u^{\prime }\neq u}{\left|h_{u}w_{u^{\prime }}\right|}^{2}$ and the BS interference term to $I_{g}={\left|h_{u}w_{g}\right|}^{2}$. The resulting modified ${\mathrm{SINR}}_{u}$ expression is given by

(A7) $${\mathrm{SINR}}_{u}^{\left(\mathrm{coupled}\right)}=\frac{{\left|h_{u}w_{u}\right|}^{2}}{I_{c}+\alpha \cdot {\left|h_{u}w_{u}\right|}^{2}+I_{g}+\sigma_{u}^{2}}=\frac{\varepsilon_{0}}{1+\alpha \cdot \varepsilon_{0}}$$

where $\varepsilon_{0}$ denotes the upper bound of SINR in the absence of coupling. It can be observed that as the coupling coefficient α increases, the actual SINR decreases.

As previously stated, we substitute $\sum_{c=1}^{C}\zeta_{c}^{2}{\left|a^{H}(\varphi_{c})w_{u}\right|}^{2}=\beta \cdot \sum_{s=1}^{S}\zeta_{s}^{2}{\left|a^{H}(\varphi_{s})w_{s}\right|}^{2}$ into the SCNR expression and set the clutter basis term as $C_{0}=\sum_{c=1}^{C}\zeta_{c}^{2}{\left|a^{H}(\varphi_{c})w_{s}\right|}^{2}+\sigma_{s}^{2}$, and the corrected SCNR can be formally expressed as

(A8) $${\mathrm{SCNR}}^{\left(\mathrm{coupled}\right)}=\frac{\sum_{s=1}^{S}\zeta_{s}^{2}{\left|a^{H}(\varphi_{s})w_{s}\right|}^{2}}{\beta \cdot \sum_{s=1}^{S}\zeta_{s}^{2}{\left|a^{H}(\varphi_{s})w_{s}\right|}^{2}+C_{0}}=\frac{\eta_{0}}{1+\beta \cdot \eta_{0}}$$

where $\eta_{0}$ denotes the upper bound of SCNR in the absence of coupling. It can be observed that as the coupling coefficient β increases, the actual SCNR decreases.

(iii) Analysis of the conditions defining the Pareto frontier

The Pareto frontier of the ISAC system is defined as the set of optimal performance points for which an improvement in SINR cannot be achieved without a corresponding degradation in SCNR, and vice versa. The analytical expression of the Pareto frontier must be derived in conjunction with power constraints to characterize the coupling performance boundary under such constraints, thereby enabling the precise formulation of the trade-off relationship between SINR and SCNR.

The communication total power is defined as $P_{c}=\sum_{u=1}^{U}\|w_{u}{\|}^{2}$, and the sensing total power is given by $P_{s}=\sum_{s=1}^{S}\|w_{s}{\|}^{2}$, satisfying the constraint $P_{c}+P_{s}=P$, where the total transmit power is fixed. As derived from leader-layer optimization in Section 3.1, in the absence of coupling, the SINR upper bound $\varepsilon_{0}$ is proportional to $P_{c}$, and it indicated that higher communication power leads to a higher SINR limit. Similarly, according to follower-layer optimization in Section 3.2, when no coupling exists, the SCNR upper bound $\eta_{0}$ is proportional to $P_{s}$, implying that increased sensing power results in a higher SCNR limit. By substituting the coupled modified ${\mathrm{SINR}}_{u}^{\left(\mathrm{coupled}\right)}$ and ${\mathrm{SCNR}}^{\left(\mathrm{coupled}\right)}$ expressions, we obtain $\varepsilon_{0}=k_{u}\cdot P_{c}$ and $\eta_{0}=k_{s}\cdot P_{s}$ ($k_{u},k_{s}>0$ is the power-performance conversion coefficient, which is determined by the channel gain and the number of antennas). Therefore, the coupled modified ${\mathrm{SINR}}_{u}^{\left(\mathrm{coupled}\right)}$ and ${\mathrm{SCNR}}^{\left(\mathrm{coupled}\right)}$ can be given by

(A9) $${\mathrm{SINR}}_{u}^{\left(\mathrm{coupled}\right)}=\frac{k_{u}P_{c}}{1+\alpha k_{u}P_{c}}$$

(A10) $${\mathrm{SCNR}}^{\left(\mathrm{coupled}\right)}=\frac{k_{s}(P-P_{c})}{1+\beta k_{s}(P-P_{c})}$$

Subsequently, we eliminate the intermediate variable $P_{c}$; the analytical equation of the Pareto frontier under coupling effects can be derived by

(A11) $$\frac{{\mathrm{SINR}}_{u}^{\left(\mathrm{coupled}\right)}}{\alpha k_{u}{\mathrm{SINR}}_{u}^{\left(\mathrm{coupled}\right)}-k_{u}}+\frac{{\mathrm{SCNR}}^{\left(\mathrm{coupled}\right)}}{\beta k_{s}{\mathrm{SCNR}}^{\left(\mathrm{coupled}\right)}-k_{s}}=P$$

Through the analytical expression of the Pareto frontier, a conclusion can be drawn:

When α=β=0 (uncoupled case), the frontier equation simplifies to ${\mathrm{SINR}}_{u}^{\left(\mathrm{coupled}\right)}/k_{u}+{\mathrm{SCNR}}^{\left(\mathrm{coupled}\right)}/k_{s}=P$, representing a linear efficiency frontier. This implies that SINR and SCNR can be perfectly balanced without any performance loss. When α>0 or β>0 (coupled case), the frontier equation describes a nonlinear curve. The greater the ratio α/β, the more pronounced the contraction of the frontier toward the origin.
